# Magnetic Resonance Imaging Features and Clinical Findings in Pediatric Idiopathic Intracranial Hypertension: A Case–Control Study

**DOI:** 10.3390/life11060487

**Published:** 2021-05-27

**Authors:** Aubrey L. Gilbert, Jennifer Vaughn, Sarah Whitecross, Caroline D. Robson, David Zurakowski, Gena Heidary

**Affiliations:** 1 Department of Ophthalmology, Boston Children’s Hospital, Harvard Medical School, Boston, MA 02115, USA; Aubrey.L.Gilbert@kp.org (A.L.G.); sarah.whitecross@childrens.harvard.edu (S.W.); 2 Department of Ophthalmology, Massachusetts Eye & Ear Infirmary, Harvard Medical School, Boston, MA 02115, USA; 3 Department of Radiology, Boston Children’s Hospital, Harvard Medical School, Boston, MA 02115, USA; jvaughn2@phoenixchildrens.com (J.V.); caroline.robson@childrens.harvard.edu (C.D.R.); 4 Department of Radiology, Phoenix Children’s Hospital, Phoenix, AZ 85016, USA; 5 Department of Anesthesiology, Boston Children’s Hospital, Harvard Medical School, Boston, MA 02115, USA; david.zurakowski@childrens.harvard.edu

**Keywords:** pediatric idiopathic intracranial hypertension, pediatric pseudotumor cerebri, papilledema

## Abstract

The purpose of this study is to identify salient magnetic resonance imaging (MRI) findings of pediatric IIH, to determine the relevance of these findings with regard to disease pathogenesis, and to relate these findings to the clinical presentation towards identification of risk factors of disease. A retrospective, a case–control study of 38 pediatric patients with and 24 pediatric patients without IIH from the ophthalmology department at a tertiary care center was performed. Clinical data, including ophthalmic findings and lumbar puncture results, were recorded. Neuroimaging, including both MRI and magnetic resonance venography (MRV), was evaluated for perioptic subarachnoid space diameter enlargement, posterior globe flattening, optic nerve head protrusion, empty or partially empty sella turcica, dural venous sinus abnormalities, skull base crowding, and prominent arachnoid granulations. Compared with controls, IIH patients had larger perioptic subarachnoid space diameters, higher incidences of posterior globe flattening, protrusion of the optic nerve heads, an empty sella turcica, and dural venous sinus abnormalities. A perioptic subarachnoid space diameter of ≥5.2 mm was identified as an independent predictor of IIH (*p* < 0.001) with sensitivity of 87% and specificity of 67%. Several significant MRI findings in pediatric IIH were identified. Using a model that uniquely incorporated clinical and MRI findings at presentation, we provide a framework for risk stratification for the diagnosis of pediatric IIH which may be utilized to facilitate diagnosis. Future prospective work is needed to further validate the model developed in this study.

## 1. Introduction

Pediatric patients with idiopathic intracranial hypertension (IIH) have the potential for profound, irreversible vision loss from papilledema [1,2]. The development of diagnostic tools which would accurately identify pediatric patients who may have IIH continue to be sought. In adults with idiopathic intracranial hypertension, patients have been shown to harbor characteristic findings on magnetic resonance imaging (MRI) which are suggestive of elevated intracranial pressure, and therefore which facilitate in diagnosis [3]. Studies evaluating the relevance of these signs of IIH in pediatric patients are limited [4,5,6,7,8,9]. Although there have been non-invasive methods which have been utilized such as ocular ultrasonography to evaluate for the presence of elevated intracranial pressure, the relationship between the clinical presentation of IIH and neuroimaging findings has not been explored in great detail to determine their relevance as a diagnostic tool for pediatric IIH [10,11].

Previous work has suggested that features that may occur in pediatric IIH include enlargement of the optic nerve sheath diameters, protrusion of the optic nerve heads, and flattening of the posterior aspects of the globes [4,5,6,7,8,9]. Those studies have not investigated the relevance of these findings in the context of the clinical presentation of pediatric IIH, however. Further, the incidence and relevance of findings on magnetic resonance venography (MRV), namely the presence of dural sinus abnormalities and their role in the pathogenesis of IIH, have not been extensively examined in pediatric patients, although transverse sinus stenosis is increasingly being implicated as a contributing factor in adult IIH [12]. In pediatric patients, further investigation is warranted to clarify whether dural venous sinus abnormalities are causative as has been suggested in some cases of adult IIH, or conversely, secondary, indirect signs of elevated intracranial pressure.

The purpose of this study is to characterize salient MRI findings of pediatric IIH and to relate these findings to the clinical presentation towards determining risk factors of pediatric IIH.

## 2. Materials and Methods

### 2.1. Study Design

This study is a retrospective review of clinical data and MRI exams, comparing IIH patients with papilledema to pediatric controls. This study was approved by our Institutional Review Board and conducted in compliance with the Health Insurance Portability and Accountability Act and the tenets of the Declaration of Helsinki.

### 2.2. Patient Selection

Inclusion criteria for IIH patients were: (a) age ≤ 18 years; (b) a prior diagnosis of IIH among patients seen between 2000 and 2011 in the department of ophthalmology based on accepted diagnostic criteria for pediatric IIH [13] with documentation of papilledema on eye examination; (c) lack of dural sinus thrombosis, intracranial mass lesion, or other underlying etiology on neuroimaging known to cause elevated intracranial pressure; (d) a lumbar puncture opening pressure ≥ 28 cm H_2_O and normal cerebrospinal fluid constituents; and (e) no other significant medical issues. The date of diagnosis was the date in which all diagnostic criteria were fulfilled (typically at the time of lumbar puncture), and the imaging measurements detailed below were performed on neuroimaging closest to the date of diagnosis. All cases were reviewed for inclusion by two of the authors, both fellowship trained in pediatric and neuro-ophthalmology (AG and GH). Data for IIH and control patients regarding demographic details, symptoms and signs of idiopathic intracranial hypertension including headache, transient visual obscurations, pulsatile tinnitus, the presence or absence of diplopia, the presence of a 6th nerve palsy, and the presence of papilledema at presentation were abstracted from review of the medical record.

Control patients were found by searching a radiology database of individuals up to age 18 having prior high-resolution MRIs and MRVs that were interpreted as normal. Patients may have been referred for neuroimaging for the symptom of headaches, and were excluded from the study if any significant medical issues were found. From this group, patients were selected for analysis who had documentation of a normal comprehensive eye examination (performed within three months of the time of their imaging) showing no optic nerve edema. All eligible controls who met the above criteria were included for the study.

### 2.3. Quantitative Assessment of Neuroimaging

Measurements of the perioptic subarachnoid space diameter were made on the axial T2-weighted fast spin-echo (FSE) sequence of the brain (repetition time (TR) = 11,730 ms; echo time (TE) = 91 ms; 2.5 mm thickness; 0 mm spacing; field of vision (FOV) = 18.2 cm × 20 cm; NEX 2) and, when available, on the fat-suppressed T2-weighted sequence of the orbits (TR 3650 ms; TE 94 ms; 3.0 mm thickness; 0 mm spacing; FOV 16 cm × 17.6 cm; NEX 3). Imaging was performed on either a 3T MR (Siemens TrioTim or Skyra, Erlangen, Germany) or 1.5T MR (GE Signa Twin, Fairfield, CA, USA) scanner. Enlargement of the optic nerve sheath diameter was evaluated by measuring the outer diameter of the perioptic subarachnoid space. The eye in which the optic nerve and sheath were most clearly seen was used per patient as it has been demonstrated that there is not a statistically significant difference in optic nerve sheath diameter when within eyes comparisons are made for the same subject [14]. Measurements were made on the axial T2 FSE sequence and, when available, on the T2 high-resolution fat-suppressed dedicated orbital sequence. The diameter of the optic nerve sheath was measured in the axial plane, 4 mm posterior to the dorsal aspect of the globe, perpendicular to the plane of the optic nerve in accordance with previously published work [15,16].

### 2.4. Qualitative Assessment of Neuroimaging

The axial T2-weighted FSE sequence (parameters as previously described), the sagittal T1-weighted magnetization prepared rapid gradient echo (MPRAGE) sequence (TR 2530 ms; TE 1.7 ms; 1.0 mm thickness; FOV 22 cm × 24.2 cm), and the coronal 2-dimensional time of flight magnetic resonance venography (2D TOF MRV) (TR 20 ms; TE 5.6 ms; 2.0 mm thickness; 0 mm spacing; FOV 18.2 cm × 20 cm) or sagittal 2D TOF MRV (TR 40 ms; TE 4.9 ms; 1.6 mm thickness; 0 mm spacing; FOV 24 cm × 26.4 cm) in addition to the 3D rotating maximum intensity projection (MIP) reformatted images of the MRV were assessed. Our aim was to verify the presence or absence of a number of findings that have been demonstrated previously in the setting of intracranial hypertension as well as to observe any additional novel findings that may suggest alterations in cerebrospinal fluid (CSF) dynamics.

Assessment of posterior globe flattening was made by notation of a change in normal contour. Assessment for protrusion of the optic papilla was determined by whether or not the optic nerve head could be seen protruding into the posterior aspect of the vitreous. Assessment of the presence of arachnoid granulations was made on the axial T2 sequence of the brain by observation of protrusions of sharply defined CSF signal intensity foci in contiguity with the extra axial space into the dural venous sinuses or into the inner table of the calvarium with bony remodeling. The presence of an empty or partially empty sella turcica was made on the sagittal T1-weighted MPRAGE sequence by observing the sella filled with CSF signal intensity fluid and flattening of the pituitary gland along the floor of the sella with the infundibulum traversing the space, with or without sella enlargement. Determination of crowding at the foramen magnum was made based on the axial T2-weighted FSE sequence, demonstrating effacement of the CSF space at the level of the foramen magnum, mass effect on the dorsal medulla and upper cervical cord, and kinking of the cervicomedullary junction.

**Figure 1:**
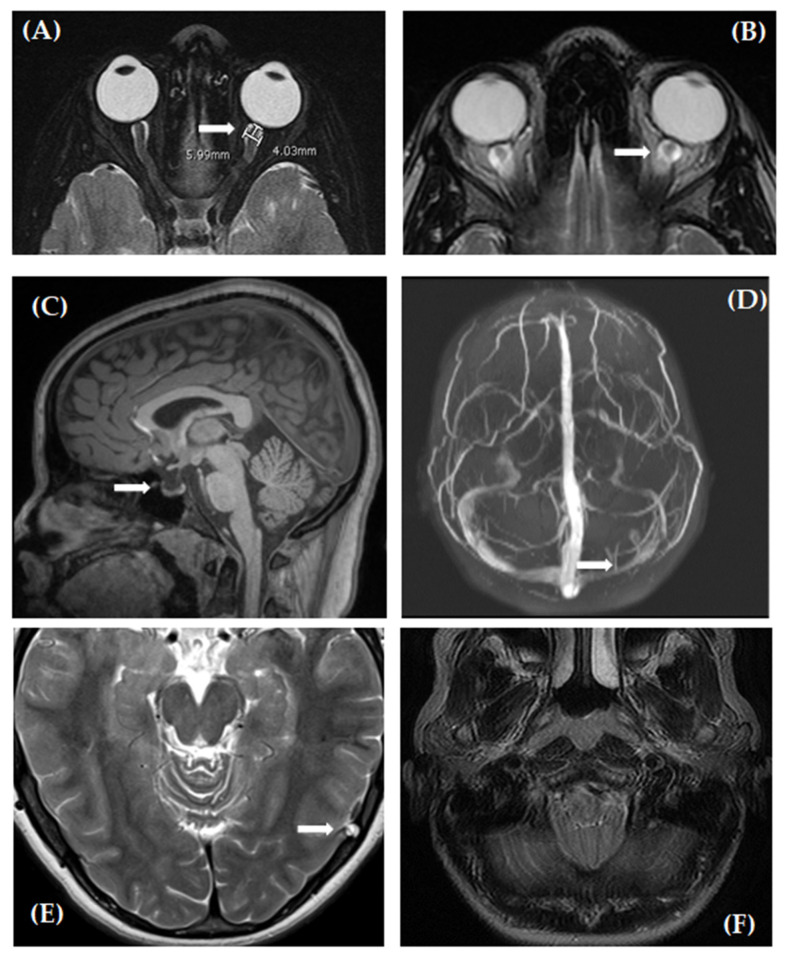
Neuroimaging examples from IIH patients. Axial fat-suppressed T2-weighted image of the orbits (**A**) demonstrates intraocular protrusion of the optic nerve head bilaterally and distension of the perioptic subarachnoid space, as measured 4 mm posterior to the globe perpendicular to the long axis of the optic nerve, on the left (arrow). In this example, the diameter measured 5.99 mm as shown. Axial T2-weighted image of the orbits (**B**) demonstrates flattening of the posterior contour of the globes bilaterally. The perioptic subarachnoid space is distended (arrow). Sagittal T1-weighted MPRAGE image of the brain (**C**) demonstrates a partially empty sella turcica (arrow). Maximum intensity projection (MIP) image from a coronal 2D TOF MRV (**D**) demonstrates segmental stenosis or hypoplasia of the left transverse sinus (arrow). Axial T2-weighted image of the brain (**E**) demonstrates a prominent arachnoid granulation (arrow). Axial T2-weighted image of the brain (**F**) at the level of the foramen magnum demonstrates cerebellar tonsillar ectopia with crowding.

Dural venous sinus abnormalities were assessed on the source imaging of the 2D MRV. The sinuses were considered abnormal if any of the following features were noted: focal or diffuse long segment sinus narrowing, atresia or absence of a sinus, and/or a flow gap within the transverse sinus. If none of these features were present, the sinuses were interpreted to be normal. The presence of a nondominant transverse sinus was not considered an abnormality. The findings on the source images were corroborated on MIP images. Representative images for the MRI findings that were evaluated in this study are shown in Figure 1.

All imaging was reviewed by one of the authors, a board-certified pediatric neuroradiologist who was blinded to the clinical data (JV). Given that the anatomy of the transverse and sigmoid sinuses can be variable and true stenosis may be more difficult to discern, MRV imaging was reviewed by two authors both board-certified in pediatric neuroradiology (JV and CR) with consensus agreement on the presence or absence of any abnormalities related to the dural venous sinuses.

### 2.5. Statistical Analysis

Univariate analysis included a comparison of IIH patients and controls with respect to demographics (age, gender, body mass index), clinical features (headache, pulsatile tinnitus, visual obscurations, diplopia/sixth nerve palsy), and MRI findings (perioptic subarachnoid space diameter, presence or absence of posterior globe flattening, optic nerve protrusion, empty or partially empty sella, dural venous sinus abnormalities, skull base crowding, prominent arachnoid granulations) using the unpaired Student’s t-test for continuous (normally distributed) data and Fisher’s exact test (version 7.0, nQuery Advisor, Statistical Solutions, Cork, Ireland) for binary proportions. Statistical analysis was performed by one of the authors (DZ), director of biostatistics at our institution. Post hoc analysis demonstrated that sample sizes of 38 patients with IIH and 24 controls provided 80% statistical power to detect 20% differences in clinical and MRI variables between the two groups using Fisher’s exact test. Two-tailed values of *p* < 0.05 were considered statistically significant. Statistical analysis was performed using IBM SPSS Statistics (version 23.0, IBM, Armonk, NY, USA).

For comparing the perioptic subarachnoid space diameter as a continuous MRI predictor variable, post hoc analysis with G*Power 3.1 software (Dusseldorf, Germany) confirmed that a minimum sample size of 24 patients in each study group (IIH, Controls) would achieve 80% statistical power to detect a mean difference of 0.5 mm, assuming a standard deviation of 0.6mm (effect size: 0.83) using a Student’s t-test for two independent groups and a two-sided alpha level of 0.05 [17].

Since univariate analysis revealed that IIH patients have significantly larger perioptic subarachnoid space diameters, we applied receiver operating characteristic curve analysis to assess the utility of the perioptic subarachnoid space diameter in differentiating between patients with IIH and controls with area under the curve to measure the predictive accuracy with a corresponding 95% CI and the Youden J-index to identify the optimal cutoff value for maximizing the relationship between sensitivity and specificity [18].

Based on the two clinical features and five MRI findings at presentation that were significantly associated with IIH in the univariate analysis, we computed a total number of these variables for each patient ranging from 0 to all 7 and used receiver operating characteristic (ROC) curve analysis to assess the diagnostic accuracy of this score to differentiate between the pediatric IIH and control groups, with area under the curve (AUC) as a measure of predictive accuracy and the Youden J-index to identify the optimal cutoff value that maximizes the combination of sensitivity and specificity [18]. We also compared the median and interquartile range of the total number of predictive variables (from 0 to 7) for the IIH and controls groups using the Mann–Whitney *U*-test and derived the predicted probability of IIH based on the total number of the 7 variables using logistic regression analysis.

## 3. Results

Neuroimaging and clinical findings of 38 IIH patients and 24 control patients were evaluated. Demographic features and MRI findings of the study cohort are shown in Table 1. The IIH patient and control populations did not differ significantly by age, gender, or body mass index. In terms of presenting clinical symptoms and ophthalmological signs on examination, the IIH patients had higher incidences of clinical findings associated with IIH compared with the control patients, including reports of pulsatile tinnitus and diplopia as detailed in Table 1. There was no significant difference between the two groups in incidence of headache. The presence of a sixth nerve palsy was only found in IIH patients. Where indicated, the denominator less than the n indicates that complete data were not available for the specific parameter.

As shown in Table 1, enlarged perioptic subarachnoid space diameter, posterior globe flattening, optic nerve protrusion, partially empty sella turcica, and dural venous sinus abnormalities were significantly associated with IIH.

We explored a number of MRI findings to determine their association with pediatric IIH which have not previously been examined in great detail. Of the MRI signs evaluated, there was no significant difference between IIH patients and controls in the incidence of skull base crowding or prominent arachnoid granulations (Table 1).

**Figure 2:**
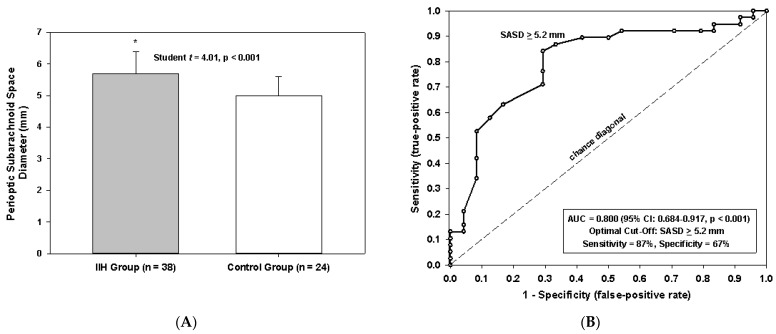
Evaluation of subarachnoid space diameter comparing pediatric IIH patients with controls. (**A**) Mean perioptic SAS diameter for both cohorts. Bars illustrate significantly larger mean perioptic SAS diameters among patients with IIH compared with controls (* *p* < 0.001, Student’s *t*-test). Error bars denote standard deviations. (**B**) Receiver operating characteristic curve shows accuracy of SAS diameter as judged by the area under the curve of 0.800 in predicting IIH (* *p* < 0.001), with the optimal cutoff identified as SAS diameter ≥5.2 mm (value of SAS farthest from chance diagonal) for maximizing the relationship between sensitivity and specificity. SAS, subarachnoid space; SASD, subarachnoid space diameter.

IIH patients had significantly larger mean perioptic subarachnoid space diameters (5.7 mm vs. 5.0 mm, *p* < 0.001) (Figure 2A). Receiver operating characteristic curve analysis evaluating enlarged perioptic subarachnoid space diameter alone for predicting IIH was performed and found to peak at 5.2 mm, with a sensitivity of 87% and a specificity of 67% (Figure 2B).

Informed by the univariate analysis shown in Table 1, we sought to combine the seven clinical and neuroimaging findings significantly associated with IIH (*p* < 0.05) into an aggregate score for risk stratification or predicted probability for a diagnosis of IIH shown in Table 2. As above in Table 1, the variables significantly associated with IIH included two clinical variables (pulsatile tinnitus and diplopia) and five MRI findings (enlarged perioptic SAS diameter ≥ 5.2 mm, posterior globe flattening, optic nerve head protrusion, empty sella turcica, and transverse sinus narrowing). The 38 IIH patients had a significantly higher score than the 24 controls: median (interquartile range; minimum–maximum): 4 (3–5; 1–7) versus 1 (0–1; 0–3), *p* < 0.0001). Furthermore, the total score was a highly significant predictor of IIH with area under the ROC curve of AUC = 0.952 (95% confidence interval: 0.903–1.000, *p* < 0.0001). Thus, these seven variables which were significantly more common in pediatric IIH, including the larger SAS diameter, together appeared to provide excellent diagnostic predictive accuracy of IIH. The Youden J-index in ROC analysis identified an optimal predictive cutoff value that differentiated pediatric IIH from controls as 3 or higher in the total score or number of variables (ranging from 0 to 7). This cutoff has a sensitivity of 82% and a specificity of 96%.

## 4. Discussion

We have sought to clarify which MRI findings that have been implicated in adults with IIH are present and relevant in a pediatric cohort of IIH patients compared with pediatric controls and to relate these factors to the clinical presentation of the disease. Several highly sensitive MRI findings in pediatric IIH were identified. Compared with controls, IIH patients had larger perioptic subarachnoid space diameters and higher incidences of flattening of the posterior globes, protrusion of the optic nerve heads, and empty or partially empty sella turcica. The finding of enlargement of the perioptic nerve sheath diameters in IIH patients is consistent with prior studies using smaller cohorts of pediatric IIH patients [4,5,6,7,8,9]. Our analysis extends this observation further to define a cutoff point (>5.2 mm) which, when measured according to the technique outlined above, has a high sensitivity and specificity for papilledema in pediatric IIH and may serve as a useful diagnostic tool. Further, by analyzing the MRI findings with the clinical data for our patients, we were able to identify characteristics of the presentation towards predicting, with higher probability, the presence of IIH.

We evaluated a number of MRI aspects that have not been examined previously in children but that may have an association with elevated intracranial pressure in pediatric IIH. We established that the following findings did not appear to be significantly associated: crowding of the foramen magnum or prominent arachnoid granulations.

Interestingly, IIH patients had a higher incidence of dural venous sinus abnormalities, which has not been previously investigated or reported in children. It is unclear whether this finding could be secondary to increased intracranial pressure or could actually underlie the basic pathophysiology of the disease [19]. In adults, endovascular stenting in the setting of transverse sinus stenosis is being performed to treat IIH [12]. Our findings highlight the need to further investigate the extent to which dural sinus abnormalities contribute to pediatric IIH.

Our study is limited by its retrospective nature. Not all clinical data were available for every patient. With respect to neuroimaging, there is the possibility of errors being introduced by manual quantitative measurements. Furthermore, slight differences in measurements performed on different MRI scanners may exist. Review of a larger number of patients and controls could allow for further subgroup analyses. Since some prior studies have suggested fundamental differences in IIH in pre- and post-pubertal individuals, [1,2] it would be interesting to perform subgroup analyses by, for example, Tanner staging. Future prospective studies to evaluate this would be valuable.

## 5. Conclusions

Our work highlights salient MRI findings in pediatric IIH that, when coupled with presenting clinical symptoms, are highly predictive of disease. The use of such an approach may serve to more effectively and promptly identify at-risk patients, thereby protecting pediatric patients from the potential of profound and irreversible visual impairment from IIH. This may be particularly relevant in equivocal cases where the diagnosis is less certain. Compared with healthy controls, pediatric IIH patients had larger perioptic subarachnoid space diameters; a diameter measuring ≥ 5.2 mm was identified to be an independent predictor of IIH (*p* < 0.001) with sensitivity of 87% and specificity of 67%. Using a model that uniquely incorporated clinical and MRI findings at presentation, we provide a framework for risk stratification for the diagnosis of pediatric IIH, which may be utilized to facilitate diagnosis. Future prospective work is needed to further validate the model developed in this study.

## Figures and Tables

**Table 1:** Demographics, clinical data, and MRI signs of IIH and control groups.

Characteristic	IIH Patients (n = 38)	Control Group (n = 24)	Univariate *p*-Value
**Demographics, Clinical Symptoms**			
Age, years	11.9 ± 4.7	13.9 ± 3.2	0.071
Female gender (%)	24 (63)	17 (71)	0.591
Body mass index, kg/m^2^	27.2 ± 9.9	23.5 ± 7.1	0.119
Lumbar puncture pressure, cm H_2_O	40 ± 10	NP	-
Sixth nerve palsy (%)	6 (16)	0 (0)	0.073
Headache (%)	27/37 (73)	13 (54)	0.171
Pulsatile tinnitus (%)	11/33 (33)	0 (0)	0.001
Transient visual obscurations (%)	7/28 (25)	1 (4)	0.056
Diplopia (%)	10/33 (30)	1 (4)	0.017
**MRI Signs**			
Perioptic subarachnoid space diameter, mm	5.7 ± 0.7	5.0 ± 0.6	<0.001
Posterior globe flattening (%)	28 (74)	0 (0)	<0.001
Optic nerve protrusion (%)	17 (45)	0 (0)	<0.001
Empty sella (%)	20 (53)	2 (8)	<0.001
Dural Venus Sinus Abnormalities (%)	20/29 (69)	9 (38)	0.029
Foramen magnum crowding (%)	1 (3)	1 (4)	1.000
Prominent arachnoid granulations (%)	3 (8)	5 (21)	0.242

Continuous data are mean ± standard deviation. NP, not performed.

**Table 2:** Predicted probability of IIH based on seven significant univariate variables (*p* < 0.05).

Total Score per Patient	Predicted Probability IIH (%)
0	3
1	17
2	55
3	93
4	99
5	100
6	100
7	100

## Data Availability

The data presented in this study are available on request from the corresponding author. The data are not publicly available due to privacy.
